# GABA Receptors on Orexin and Melanin-Concentrating Hormone Neurons Are Differentially Homeostatically Regulated Following Sleep Deprivation^1^,^2^,^3^

**DOI:** 10.1523/ENEURO.0077-16.2016

**Published:** 2016-06-09

**Authors:** Hanieh Toossi, Esther del Cid-Pellitero, Barbara E. Jones

**Affiliations:** Department of Neurology and Neurosurgery, Montreal Neurological Institute, McGill University, Montreal, Quebec, Canada H3A 2B4

**Keywords:** GABA_A_R, GABA_B_R, homeostasis, mice, waking

## Abstract

Though overlapping in distribution through the hypothalamus, orexin (Orx) and melanin-concentrating hormone (MCH) neurons play opposite roles in the regulation of sleep–wake states. Orx neurons discharge during waking, whereas MCH neurons discharge during sleep. In the present study, we examined in mice whether GABA_A_ and GABA_B_ receptors (Rs) are present on Orx and MCH neurons and might undergo differential changes as a function of their different activities following sleep deprivation (SD) and sleep recovery (SR). Applying quantitative stereological image analysis to dual-immunofluorescent stained sections, we determined that the proportion of Orx neurons positively immunostained for GABA_A_Rs was significantly higher following SD (∼48%) compared with sleep control (SC; ∼24%) and SR (∼27%), and that the luminance of the GABA_A_Rs was significantly greater. In contrast, the average proportion of the MCH neurons immunostained for GABA_A_Rs was insignificantly lower following SD (∼43%) compared with SC (∼54%) and SR (56%), and the luminance of the GABA_A_Rs was significantly less. Although, GABA_B_Rs were observed in all Orx and MCH neurons (100%), the luminance of these receptors was differentially altered following SD. The intensity of GABA_B_Rs in the Orx neurons was significantly greater after SD than after SC and SR, whereas that in the MCH neurons was significantly less. The present results indicate that GABA receptors undergo dynamic and differential changes in the wake-active Orx neurons and the sleep-active MCH neurons as a function of and homeostatic adjustment to their preceding activity and sleep–wake state.

## Significance Statement

The activity of single neurons is regulated in a homeostatic manner such that prolonged activity results in decreased excitability. Orexin neurons discharge during waking, whereas MCH neurons do so during sleep. Here, we examined whether the inhibitory GABA receptors (Rs) on Orexin and MCH neurons would change differentially as a function of their different activities following sleep deprivation and sleep recovery. Whereas GABA_A_R and GABA_B_R immunostaining appeared to increase on Orexin neurons, it appeared to decrease on MCH neurons after sleep deprivation relative to sleep control and sleep recovery. GABA receptors thus undergo differential changes on Orx and MCH neurons as a function of and homeostatic adaptation to their different activities during waking and sleep.

## Introduction

Orexin (Orx) and melanin-concentrating hormone (MCH) peptides are contained in distinct though codistributed neurons in the hypothalamus (Bittencourt et al., 1992; Broberger et al., 1998; de Lecea et al., 1998). From multiple lines of evidence, they appear to play opposite roles in the regulation of waking and sleep. Pre-pro-Orx knock-out mice present with a syndrome of narcolepsy with cataplexy, marked by the sudden passage from waking to REM sleep with muscle atonia (Chemelli et al., 1999). Humans having narcolepsy with cataplexy have a reduced number of Orx neurons or an absence of its peptide in CSF (Peyron et al., 2000; Thannickal et al., 2000). In rats, Orx neurons fire maximally during waking and become virtually silent during sleep (Lee et al., 2005), and they express c-Fos, a marker for neuronal activity, following sleep deprivation (SD) and not sleep recovery (SR; Modirrousta et al., 2005). In contrast, MCH neurons do not fire during waking, but fire sparsely during slow-wave sleep (SWS) and maximally during REM or paradoxical sleep (PS) (Hassani et al., 2009), and they do not express c-Fos after SD but do so after SR (Verret et al., 2003; Modirrousta et al., 2005). We queried whether the different discharge profiles of the Orx and MCH neurons would be associated with different homeostatic responses of those neurons to SD.

Neuronal activity is regulated in a homeostatic manner such that increases in activity are compensated for by decreases in excitability and decreases in activity by increases in excitability (Turrigiano, 1999). These changes are mediated in part by changes in receptors to the inhibitory neurotransmitter GABA, as well as by reciprocal changes in those to the excitatory neurotransmitter, glutamate (Turrigiano et al., 1998; Kilman et al., 2002; Marty et al., 2004). With the knowledge that Orx neurons are active, whereas MCH neurons are silent during continuous waking with SD, we thus examined whether the changes in activity that occur in those neurons would be associated with differential changes in the receptors to GABA. Through *in vitro* studies, it is known that Orx neurons are hyperpolarized and inhibited by both GABA_A_ (eg, muscimol) and GABA_B_ (eg, baclofen) receptor agonists and that MCH neurons are inhibited by GABA_A_R agonists (Eggermann et al., 2003; van den Pol et al., 2004; Xie et al., 2006). We thus investigated whether homeostatic changes in response to state specific prolonged activity or absence thereof would be evident in GABA_A_R and GABA_B_R immunostaining following SD and SR in the Orx and MCH neurons.

## Materials and Methods

All procedures were done in accordance with the Canadian Council on Animal Care and were approved by the McGill University Animal Care Committee.

### Animals

Male adult mice (*n* = 25; C57BL/6, 20–25g) were received from the supplier (Charles River Laboratories) and housed individually in cages, which were maintained at an ambient temperature of 22°C, in a 12 h light/dark cycle (lights on from 7:00 A.M. to 7:00 P.M.) and they were given *ad libitum* access to water and food. Animals were maintained in their home cages for the duration of the experiment and therein recorded by video alone (VM; *n* = 13) or by video plus telemetry (VTM; *n* = 12). For telemetric recording of the electroencephalogram (EEG), a transmitter (F20-EET, Data Sciences International) was implanted subcutaneously along the flank and connected to two EEG electrodes placed symmetrically over parietal cortex and two reference electrodes placed over the cerebellum. Following surgery, the mice were allowed 1 week to recover.

### Sleep deprivation and recovery experimental procedures

As employed in another study (del Cid-Pellitero, Plavski, Mainville and Jones, unpublished observations), four experimental groups of mice were processed: (1) sleep control (SC), having undisturbed sleep and waking for 2 h from ∼2:00 to ∼4:00 P.M. (∼ZT 7–9; *n* = 7); (2) SD, being submitted to 2 h of SD from ∼2:00 to ∼4:00 P.M. (∼ZT 7–9; *n* = 6); (3) SD, being submitted to 4 h of SD from ∼12:00 to ∼4:00 P.M. (∼ZT 5–9; *n* = 5); and (4) SR, being subjected to 4 h of SD from ∼10:00 A.M. to ∼2:00 P.M. followed by 2 h SR from ∼2:00 to ∼4:00 P.M. (∼ZT 7–9; *n* = 7). SD was performed by preventing mice from going to sleep by stimulation of the whiskers with a soft paintbrush. For scoring of sleep and waking, mice were recorded by VM for behavior (*n* = 13) or by VTM for behavior with EEG (*n* = 12) using HomeCageScan software (3.0; Clever Systems). At the end of the experimental period ∼4:00 P.M. (∼ZT 9), the mice were immediately anesthetized with sodium pentobarbital (Euthanyl, 100 mg/kg; Bimeda-MTC Pharmaceutical). Brains were fixed by transcardial perfusion with 30 ml saline followed by 200 ml of 3% paraformaldehyde. The brains were removed and placed for 1 h in 3% paraformaldehyde for postfixation at 4° C, transferred to 30% sucrose solution for cryoprotection at 4° C for 2 d, and then frozen and stored at −80° C.

### Immunohistochemical processing

Brains were cut and processed in batches of two to four, which included mice from SC, SD, and/or SR groups of the same experimental session or period. Coronal sections were cut on a freezing microtome at 20 μm thickness through the diencephalon. Adjacent series of sections were collected at 200 μm intervals for immunohistochemical staining. Free-floating sections were rinsed in 0.1m trizma saline buffer, pH 7.4, and then incubated in 6% normal donkey serum buffer for 30 min and subsequently incubated overnight at room temperature in a buffer containing 1% normal donkey serum with combinations of two primary antibodies: goat anti-MCH (1:250; Santa Cruz Biotechnology, Catalog #sc-14507, RRID: AB_2166711) or goat anti-Orx (1:500; Santa Cruz Biotechnology, Catalog #sc-8070, RRID: AB_653610) with mouse anti-GABA_A_R β-chain [1:100; clone BD17, Millipore (Chemicon), Catalog #MAB 341, RRID: AB_2109419] or guinea pig anti-GABA_B_R1 [1:2500; Millipore (Chemicon), Catalog #AB1531, RRID: AB_2314472]. Both the GABA_A_R β-chain and GABA_B_R1 antibodies were produced and characterized years ago and have since been in use over many years (Fritschy and Mohler, 1995; Wan et al., 1997; Nusser et al., 1998; Bonino et al., 1999; Margeta-Mitrovic et al., 1999; Filippov et al., 2000; Straessle et al., 2003). Subsequently, sections were incubated at room temperature for 2 h in appropriate combinations of cyanine-conjugated (Cy3 or Cy5) secondary antibodies from donkey (Jackson ImmunoResearch Laboratories): Cy5-conjugated anti-goat (1:800; Catalog #705-175-147, RRID: AB_2340415) with Cy3-conjugated anti-mouse (1:1000; Catalog #715-165-150, RRID: AB_2340813), or Cy3-conjugated anti-guinea pig (1:1000; Catalog #706-165-148, RRID: AB_2340460). After rinsing the sections with trizma saline, sections were stained with green fluorescent Nissl stain (FNS; 1:2000; Molecular Probes, Catalog #N-21480) for 20 min. Finally, sections were rinsed, mounted, and coverslipped with glycerol.

### Image analysis

Triple-stained sections were viewed with a Leica DMLB microscope equipped with fluorescence filters for excitation and emission of Cy2, Cy3, and Cy5 dyes, a digital camera (Orca-R^2^, C10600-10B, Hamamatsu Photonics KK) and an *x*-*y*-*z* movement-sensitive stage. Images were acquired from three sections in each series (with 200 μm intervals between sections) through the Orx and MCH neurons in the tuberal hypothalamus using StereoInvestigator software (MicroBrightField). With the optical fractionator probe for unbiased sampling and counting, contours were first traced with a 5× objective around all the Orx or MCH neurons in each section within the lateral hypothalamus, perifornical area, dorsomedial nucleus, and/or zona incerta (Modirrousta et al., 2005). For sampling, a grid size of 250 × 150 μm^2^ was used over each contour, and for cell counting and measurements, a counting frame of 120 × 120 μm^2^ was used and placed within each rectangular space by the program. In these, multichannel image stacks were acquired under a 40× objective and were comprised by optical sections of 0.5 μm thickness through the mounted histological section of ∼15 µm thickness. Within these images, the tops of all cells located <1 µm from the surface of the section were counted, thus through 14 µm of the section within the counting frame. Across the three sections, ∼38 counting frames for Orx neurons and 59 for MCH neurons were acquired and analyzed per series. With this sampling, the average number of Orx+ cells counted across series on one side was 56.6 ± 0.70 (mean ± SEM), corresponding to an estimated total number of 1559 ± 70 Orx+ neurons within one side of the tuberal hypothalamus of the mouse. The average number of MCH cells counted was 83.96 ± 0.80, corresponding to an estimated total number of 2364 ± 86 MCH+ neurons. By moving through the *z*-plane, the double-labeling of the cells for the GABA_A_ receptors (Rs) on the membrane or GABA_B_Rs in the cytoplasm was determined. Estimated total numbers of double-labeled cells were computed for each series (GABA_A_R-Orx or GABA_A_R-MCH in 12 VTM and GABA_B_R-Orx or GABA_B_R-MCH in 13 VM) and expressed as percentage of Orx+ or MCH+ cell populations per series.

Luminance measurements were performed on the Orx+ and MCH+ cells that had been counted as positively stained for GABA_A_R or GABA_B_R in the images randomly acquired and counted using Optical Fractionator (above). So as to analyze similar numbers across groups, 8–10 double-labeled Orx+ or MCH+ cells, which were present in all animals, were analyzed per animal. The images had been acquired under the same gain and exposure for each series using an 8 bit setting of the digital camera to yield arbitrary units between 0 and 256 in the converted grayscale of the fluorescent images. To measure the luminance of the receptors, different approaches were used for the GABA_A_Rs concentrated over the plasma membrane versus the GABA_B_Rs located in the cytoplasm as well as over the membrane. For membrane GABA_A_Rs, a box of 1.5 × 0.3 μm^2^ was placed over the membrane and another over the nucleus to measure and subtract background staining in each cell. For membrane plus cytoplasmic GABA_B_Rs, a donut-shaped contour was drawn around the cytoplasm and plasma membrane, and another traced around the nucleus to measure and subtract background staining in each cell.

Cell counts and luminance measurements were analyzed between experimental groups for each cell type (Orx or MCH) and receptor (GABA_A_ or GABA_B_) using one-way ANOVA for main effect of group followed by *post hoc* paired-comparisons with Tukey’s HSD correction for differences between groups (SYSTAT Software, v13; Table 1). Given that there was no significant difference between the two SD 2 h and 4 h groups, they were combined into one SD group.

**Table 1.** Summary of statistics

Dataset	Figure	One-way ANOVA (group = 3 levels) *F* value	df; group, error	*p* value	Tukey’s HSD paired-comparisons *p* value		
					SC-SD	SC-SR	SD-SR
% Wake	1A	1032.33	2, 22	<0.001	<0.001*	<0.001§	<0.001*
% GABA_A_R+/Orx+	1B1	10.27	2, 9	0.005	0.009*	0.910	0.010*
% GABA_A_R+/MCH+	1B2	1.48	2, 9	0.270	n/a	n/a	n/a
Lum GABA_A_R:Orx+	1C1	7.43	2, 115	0.001	0.010*	0.800	0.002*
Lum GABA_A_R:MCH+	1C2	5.88	2, 117	0.004	0.010*	0.970	0.020*
% GABA_B_R+/Orx+	1D1	n/a	n/a	n/a	n/a	n/a	n/a
% GABA_B_R+/MCH+	1D2	n/a	n/a	n/a	n/a	n/a	n/a
Lum GABA_B_R:Orx+	1E1	15.93	2, 127	<0.001	<0.001*	0.970	<0.001*
Lum GABA_B_R:MCH+	1E2	27.16	2, 127	<0.001	<0.001*	0.860	<0.001*

Sections were also viewed and images acquired for this publication with an LSM 710 confocal laser-scanning microscope equipped with Ar 488 nm, He-Ne 543 nm, and He-Ne 633 nm lasers for excitation and emission of Cy2, Cy3, and Cy5 dyes. Image stacks were acquired under 63× oil objective (1.4 numerical aperture, 0.5 μm thickness for each optical section) with a 1.0 airy unit pinhole size for each channel. All figures were prepared and composed in a consistent manner for brightness and contrast across groups using Adobe Creative Suite (vCS4).

## Results

### Sleep–wake states across groups

**Figure 1.**
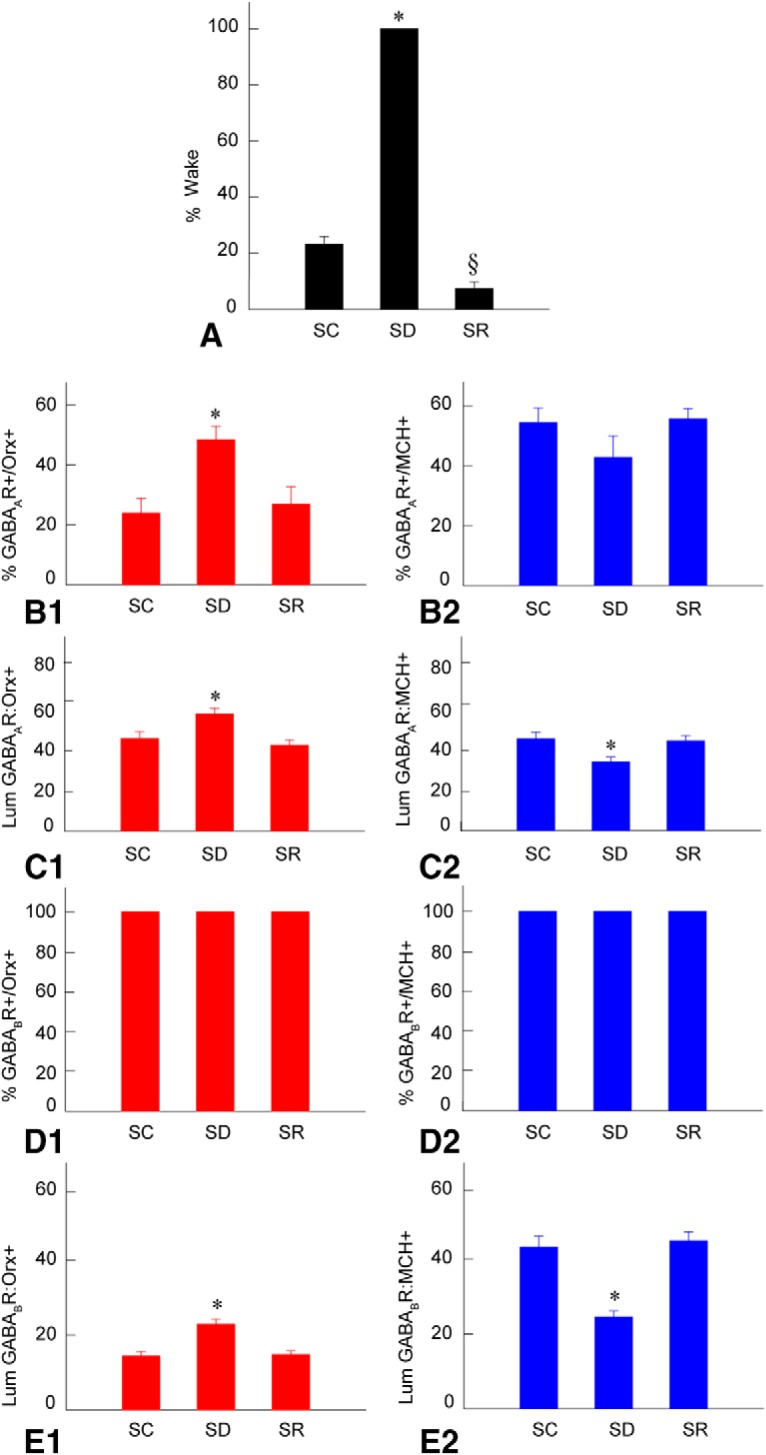
Sleep–wake states and GABA receptors in Orx and MCH neurons across groups. ***A*,** Percentage of time spent in wake during the 2 h preceding termination in SC, SD, and SR groups. Percentage wake is significantly higher in SD compared to SC and SR and significantly lower in SR compared to SC. ***B***, Proportion of Orx+ or MCH+ neurons bearing GABA_A_Rs across groups. The percentage Orx+/GABA_A_R+ was significantly greater in SD compared to SC and SR (***B1***), whereas the percentage MCH+/GABA_A_R+ neurons was insignificantly less in SD compared to SC and SR (***B2***). ***C***, Luminance of the GABA_A_R immunofluorescence on Orx and MCH neurons across groups, which was significantly increased on the Orx+ neurons (***C1***) and decreased on the MCH+ neurons (***C2***) in SD compared to SC and SR. ***D,***Proportion of the Orx (***D1***) and MCH (***D2***) neurons expressing GABA_B_Rs, which did not change across groups. ***E,***Luminance of the GABA_B_R which was significantly higher in Orx+ neurons (***E1***), and significantly lower in MCH+ neurons (***E2***) following SD compared to SC and SR. Note that the changes in GABARs on Orx neurons parallel the percentage wake, whereas those on MCH+ neurons parallel the percentage sleep across groups. *Indicates significant difference of SD relative to SC and SR (*p* < 0.05). §Indicates significant difference of SR relative to SC (*p* < 0.05), according to *post hoc* paired comparisons following one-way ANOVA (Table 1). Abbreviations: Lum, luminance.

Mice were prevented from falling asleep in the SD group (*n* = 11) and were thus continuously awake, whereas those in the SC group (*n* = 7) and SR group (*n* = 7) were awake for only a small percentage of the time during the 2 h prior to termination at ∼4:00 P.M. (Fig. 1A; Table 1). After having been previously sleep deprived, mice in the SR group were awake less or reciprocally asleep significantly more of the time (92.61 ± 2.21%, mean ± SEM) than the SC mice (76.77 ± 2.56%), indicating a homeostatic response to SD. Mice in SC and SR groups spent the majority of time in NREM sleep (66.93 ± 1.71%, *n* = 3 and 82.29 ± 4.07%, *n* = 3, respectively) and minimal time in REM sleep (9.28 ± 0.89%, *n* = 3 and 12.03 ± 0.87%, *n* = 3, respectively). Both NREM and REM sleep were significantly increased during SR relative to SC (del Cid-Pellitero, Plavski, Mainville and Jones, Unpublished observations).

### GABA_A_Rs on Orx and MCH neurons after SD and SR

**Figure 2.**
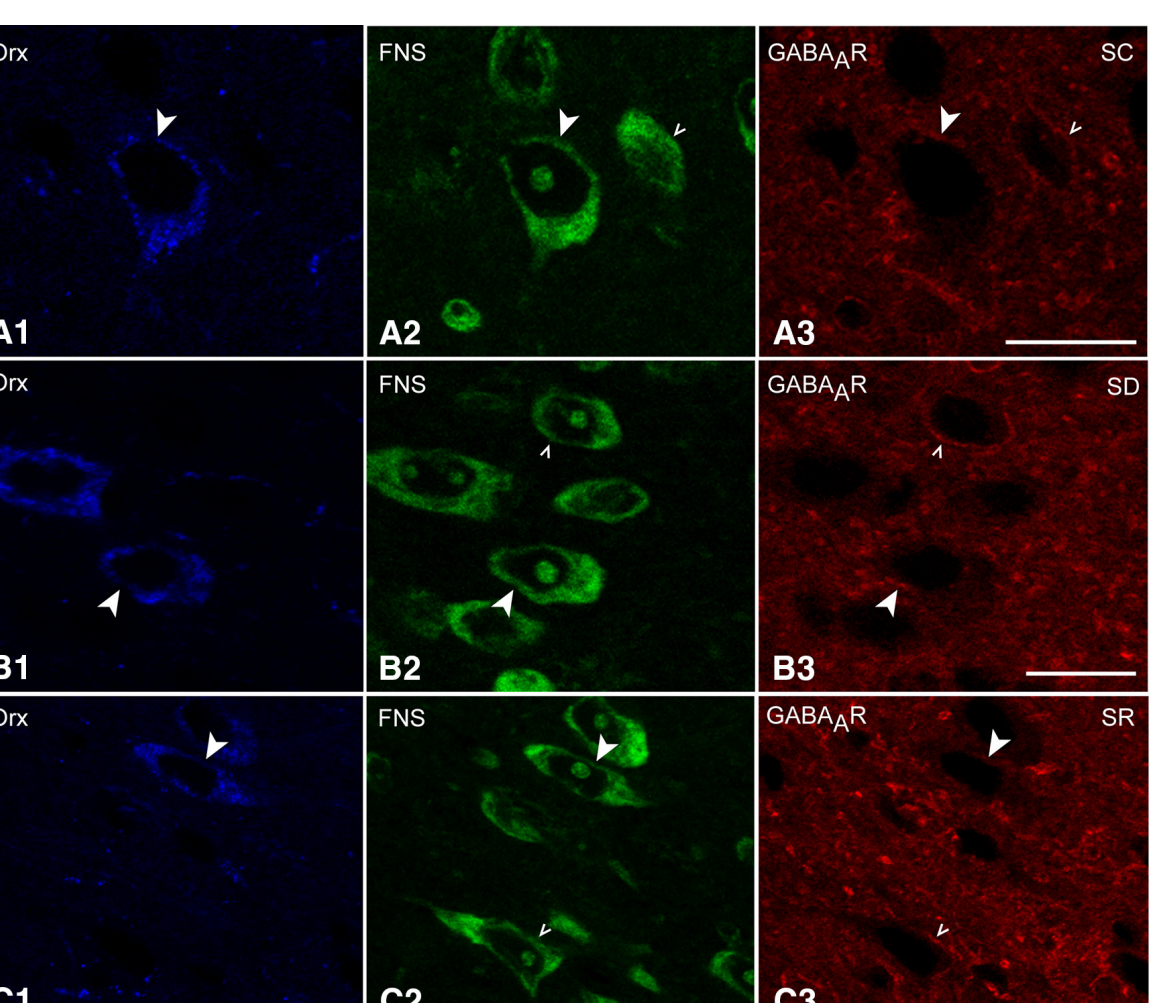
GABA_A_Rs in Orx neurons across groups. Confocal images of immunostained sections indicate that the GABA_A_R (red) was minimal on Orx+ neurons (blue, indicated by filled arrowheads) compared to that on adjacent Orx-negative neurons (stained with FNS, green, indicated by carets). ***A***, The GABA_A_R immunofluorescence was minimally visible as small clusters along a portion of the plasma membrane of an Orx+ cell body in an SC mouse, in which it was readily visible along the full membrane of an Orx-negative cell body. ***B***, The GABA_A_R staining was more visible as larger clusters along a larger portion of the membrane of an Orx+ cell in an SD mouse. ***C***, The GABA_A_R staining was similar in an SR mouse to that in SC. Scale bars, 10μm. Image thickness: ***A***, ***B***, 1500 nm; ***C***, 2000 nm.

**Figure 3.**
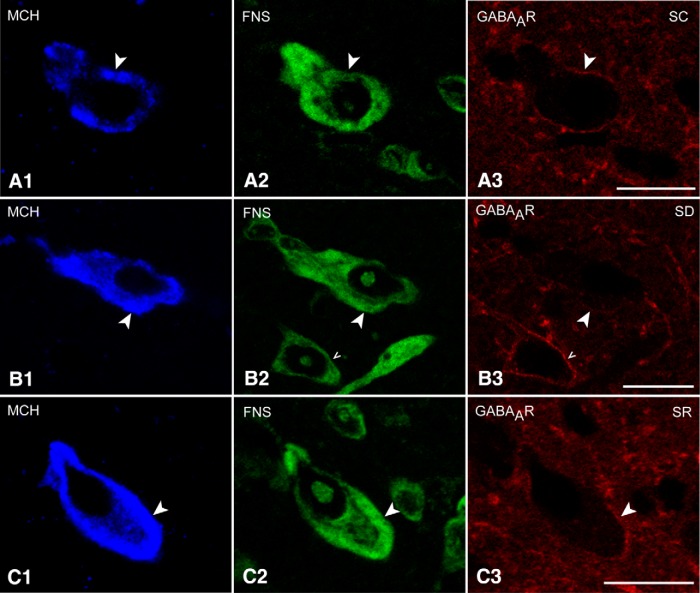
GABA_A_Rs in MCH neurons across groups. Confocal images indicate that GABA_A_R immunostaining (red) was moderate in MCH neurons (blue, indicated by filled arrowheads) though less than that in some adjacent MCH-negative neurons (stained with FNS, green, indicated by carets). ***A***, GABA_A_R immunostaining was present as clusters visible along the full plasma membrane of the cell body in an SC mouse. ***B***, GABA_A_R immunostaining was barely visible on MCH+ neurons, whereas it was prominent on adjacent MCH-negative neuron in an SD mouse. ***C***, GABA_A_R immunostaining appeared to be moderate in an SR mouse, similar to that in SC. Scale bars, 10μm. Image thickness, 500 nm.

Triple-stained sections for GABA_A_R/FNS with either Orx or MCH were analyzed to assess the presence and intensity of GABA_A_Rs on Orx and MCH neurons across the three groups (SC, SD, and SR). GABA_A_R immunostaining appeared to be located on the plasma membrane of the Orx and MCH neurons, as well as that of other surrounding neurons (Figs. 2, 3).

GABA_A_R immunostaining was minimal and patch-like on the plasma membrane of the Orx-positive (+) neurons, whereas it was often moderate and continuous on the membrane of surrounding Orx-negative neurons in the same sections (Fig. 2). Though minimal on the Orx+ neurons, the GABA_A_R immunostaining appeared to be more intense after SD compared to that after SC or SR (Fig. 2A-C). The average proportion of the Orx+ neurons, which appeared positively immunostained (+) for GABA_A_Rs on the membrane was significantly greater in the SD group (48.45 ± 4.09%, *n* = 6 mice) compared to that in the SC and SR groups (23.93 ± 3.74%, *n* = 3 and 26.93 ± 4.37%, *n* = 3, respectively; Fig. 1*B1*; Table 1). The average luminance of the GABA_A_R immunostaining on the Orx+ neurons was also significantly higher in SD (58.71 ± 2.90, *n* = 60 cells) than in SC and SR groups (46.4 ± 3.42, *n* = 30 and 43.17 ± 2.56, *n* = 28, respectively; Fig. 1*C1*; Table 1). The luminance measures did not differ between SC and SR, indicating that the GABA_A_R returned to control or baseline levels during SR.

GABA_A_R immunostaining appeared to be relatively continuous around the plasma membrane of the MCH+ neurons and somewhat more intense compared to that on Orx+ neurons (Fig. 3). Moreover, the GABA_A_R immunostaining on the MCH+ neurons appeared to be moderate in the SC and SR groups (Fig. 3*A*–*C*). In contrast, it appeared minimal following SD, even though it was prominent on surrounding MCH-negative neurons (Fig. 3*B*). The average proportion of MCH+ neurons that appeared GABA_A_R+ decreased, though not significantly so, following SD (42.86 ± 6.40%, *n* = 6 mice) compared with SC and SR (54.38 ± 3.74%, *n* = 3 and 55.7 ± 2.55%, *n* = 3 respectively; Fig. 1*B2*; Table 1). The average luminance of the GABA_A_R on the MCH+ neurons decreased significantly after SD (34.43 ± 2.45, *n* = 60 cells) compared SC and SR (45.95 ± 3.31, *n* = 30 and 44.95 ± 2.59, *n* = 30, respectively; Fig. 1*C2*; Table 1). The measures did not differ between SC and SR, indicating that the GABA_A_R returned to control or baseline levels during SR.

### GABA_B_Rs on Orx and MCH neurons after SD and SR

**Figure 4.**
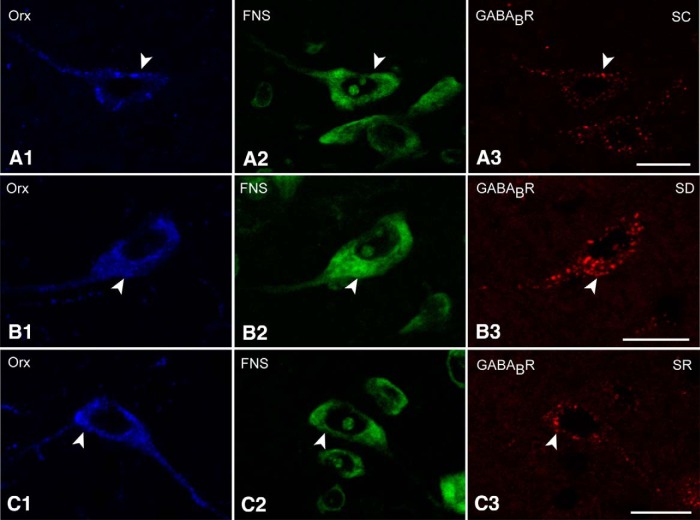
GABA_B_Rs in Orx neurons across groups. Confocal images of the GABA_B_R immunostaining (red) in Orx neurons (blue, indicated by filled arrowheads). ***A***, The GABA_B_R immunofluorescence was minimally visible as clusters over the cytoplasm of an Orx+ cell body in an SC mouse. ***B***, The GABA_B_R staining was more visible as larger clusters over the cytoplasm and partially on the plasma membrane of an Orx+ cell in an SD mouse. ***C***, The GABA_B_R staining was similar in an SR mouse to that in SC. Scale bars, 10μm. Image thickness, 1500 nm.

**Figure 5.**
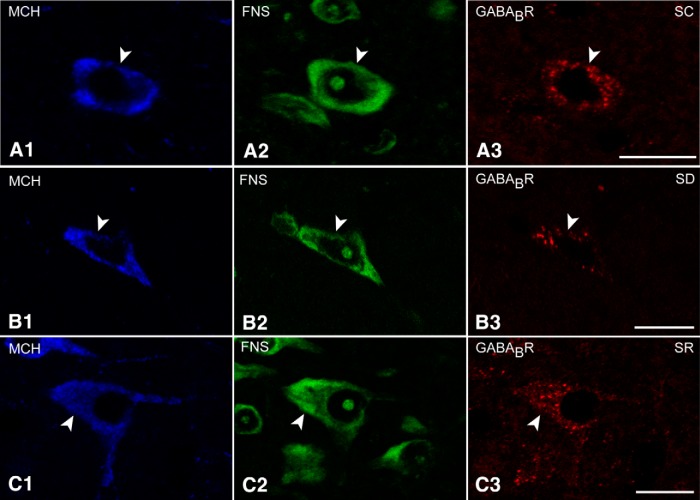
GABA_B_Rs in MCH neurons across groups. Confocal images of the GABA_B_R immunostaining (red) in MCH neurons (blue, indicated by filled arrowheads). ***A***, GABA_B_R immunostaining was present as prominent large clusters over the cytoplasm of an MCH+ neuron in an SC mouse. ***B***, The GABA_B_R immunostaining was minimally visible over the cytoplasm of an MCH+ neuron in an SD mouse. ***C***, The GABA_B_R immunostaining appeared to be prominent over the cytoplasm and near the plasma membrane of an SR mouse, similar to that in SC. Scale bars, 10μm. Image thickness: ***A***, 2000 nm; ***B***, ***C***, 1500 nm.

Triple-staining for GABA_B_R/FNS and either Orx or MCH was performed to examine the incidence of GABA_B_Rs on Orx or MCH neurons across the three groups (SC, SD, and SR). GABA_B_R immunostaining appeared to be predominantly located over the cytoplasm of the cells, whereas only minimally located on the membrane of both the Orx and MCH neurons (Figs. 4, 5).

In the Orx+ neurons, GABA_B_R immunostaining was prominent in the soma and proximal dendrites and appeared to be more dense and intense after SD than after SC and SR (Fig. 4*A*–*C*). Nonetheless, all Orx+ neurons (100%) were judged to be positively immunostained for the GABA_B_R in all mice of all groups (*n* = 4 in SC and SR groups, *n* = 5 in SD group; Fig. 1*D1*; Table 1). On the other hand, the luminance of the GABA_B_R in the Orx neurons was significantly higher following SD (22.84 ± 1.35, *n* = 50 cells) compared with SC and SR (14.36 ± 1.21, *n* = 40 and 14.76 ± 1.03, *n* = 40, respectively; Fig. 1*E1*; Table 1). The luminance did not differ between SC and SR, indicating that the GABA_B_R returned to control or baseline levels during SR.

In the MCH+ neurons, GABA_B_R immunostaining was prominent in the soma and appeared to be more dense following SC and SR than after SD (Fig. 5A–C). As for the Orx+ neurons, GABA_B_R immunostaining was nonetheless judged to be positive in all MCH+ neurons (100%) and in every group (*n* = 4 in SC and SR groups, *n* = 5 in SD group; Fig. 1*D2*; Table 1). On the other hand, the luminance of GABA_B_R immunostaining on the MCH+ neurons was significantly lower after SD (24.53 ± 1.59, *n* = 50 cells) compared with SC and SR (43.24 ± 2.87, *n* = 40 and 44.92 ± 2.33, *n* = 40, respectively; Fig. 1*E2*; Table 1). The luminance did not differ between SC and SR, indicating that the GABA_B_R returned to control or baseline levels during SR.

## Discussion

The present results indicate that GABA_A_ and GABA_B_ receptors undergo dynamic and differential changes on Orx, wake-active and MCH, sleep-active neurons as a function of SD and thus their homeostatic response to different activity changes.

### GABA_A_Rs differentially expressed as a function of sleep–wake activity

SD during the day, when mice normally sleep the majority of the time, resulted in increased GABA_A_R labeling on the membrane of the Orx neurons presumably due to prolonged activity by the Orx neurons during enforced waking, as indicated by previous c-Fos and recording studies (Lee et al., 2005; Modirrousta et al., 2005). The Orx neurons show changes in GABA_A_Rs that are parallel to those for cholinergic basal forebrain neurons following SD, when those neurons are also active, as indicated by c-Fos expression (Modirrousta et al., 2007). In addition, in the whole hypothalamus, mRNA expression for GABA_A_R (β-subunits) is also increased after SD and high activity periods (Volgin et al., 2014). In contrast, however, SD resulted in decreased GABA_A_R labeling on the membrane of MCH neurons here, presumably due to silence of the MCH neurons during waking, as indicated in previous c-Fos and recording studies (Verret et al., 2003; Modirrousta et al., 2005; Hassani et al., 2009). The changes in GABA_A_R density on the membrane seen here in the Orx and MCH neurons are similar to those described in cultured hippocampal neurons following pharmacologically induced firing and silencing, respectively (Kilman et al., 2002; Marty et al., 2004). These changes in the density of GABA_A_R clusters in the cultured neurons were moreover associated with increased versus decreased amplitude of miniature mIPSCs. An increase in membrane GABA_A_Rs was also shown to occur in hippocampal neurons *in vivo* after increased activity induced by seizures and was associated with an increase in IPSCs (Nusser et al., 1998). This increase in postsynaptic receptors appears to be the most effective way by which the magnitude of inhibitory transmission is increased (Mody et al., 1994). Somewhat similar to ours, another study in mice showed that GABA_A_R immunostaining (for the α1 subunit) was enhanced and that the sensitivity to a GABA_A_R agonist was increased along with the amplitude of IPSCs in Orx neurons following 6 h SD (Matsuki et al., 2015). The latter along with our results for the Orx neurons would appear to differ from those in rats showing increased amplitude of miniature EPSCs (mEPSCs) in Orx neurons following 4 h SD (Rao et al., 2007). However, the latter *in vitro* or *ex vivo* study was done in the presence of a GABA_A_R blocker (bicuculline) which did not allow assessment of changes in GABA_A_R currents and their potential influence on the mEPSCs. We can only assume that the increased activity by the Orx neurons during prolonged enforced waking stimulates homeostatic downscaling through increases in membrane GABA_A_Rs, which would render the neurons more susceptible and responsive to inhibition by GABA. Reciprocally, the prolonged absence of activity by the MCH neurons during prolonged waking stimulates homeostatic upscaling through decreases in membrane GABA_A_Rs, which would render them less susceptible and responsive to inhibition by GABA. The GABA_A_Rs returned to baseline levels with SR, indicating a return to normal levels of excitability and activity in both Orx and MCH cells.

### GABA_B_Rs differentially expressed as a function of sleep–wake activity

With regard to the metabotropic GABA_B_R receptor, we found that all of the Orx and MCH neurons showed positive immunostaining for that receptor across all groups. On the other hand, the density of the GABA_B_R immunostaining appeared to differ according to cell type and group. By measurement of luminance, it was found that the intensity of GABA_B_Rs in Orx neurons increased with SD presumably due to enhanced and prolonged activity with enforced waking, whereas that in MCH neurons decreased with SD, presumably due to prolonged silence. These results thus paralleled those of the GABA_A_R. In the case of the GABA_B_R, however, the immunostaining was most prominent in the cytoplasm and less evident on the plasma membrane. Although we did see staining along the membrane in some cases, we did not have adequate resolution for differentiation and systematic assessment of the membrane staining across groups. We can only assume that the different densities of GABA_B_Rs with SD reflect different expression of the receptor in homeostatic response to different activities of the Orx and MCH neurons under the abnormal conditions of sustained waking during the day when mice normally sleep the majority of the time. As with the GABA_A_Rs, the density of GABA_B_Rs returned to SC levels with SR, presumably reflecting the re-establishment of stable levels of excitability and activity during recovery sleep for both the Orx and MCH cells. Evidence from cultured hippocampus has indicated that the GABA_B_R is essential for homeostatic regulation of firing within hippocampal circuits through both presynaptic and postsynaptic mechanisms (Vertkin et al., 2015). Indeed, it has been known that genetic deletion of the GABA_B_R results in runaway excitation within these and cortical circuits resulting in seizure activity (Schuler et al., 2001), and that seizure activity is followed by increases in GABA_B_Rs in hippocampal neurons (Straessle et al., 2003). Deletion of the GABA_B_R also leads to disruption of the sleep–wake cycle in mice (Vienne et al., 2010). Fragmentation of the cycle also occurred in mice lacking GABA_B_Rs specifically on Orx neurons (Matsuki et al., 2009).

### Role of GABA receptors in neuronal homeostasis and sleep–wake regulation

GABA receptors, particularly GABA_A_Rs, have been shown to play an important role in the homeostatic regulation of neuronal excitability as a function of activity (Turrigiano, 1999). Here, we present evidence that dynamic and differential changes in both GABA_A_ and GABA_B_ receptors after SD reflect homeostatic downscaling following prolonged activity by the wake-active, Orx neurons and upscaling following inactivity by the sleep-active, MCH neurons.

Both GABA_A_ and GABA_B_ receptors, along with GABA, are known to play an important role in sleep. Most hypnotic drugs act upon the benzodiazepine binding site of the GABA_A_R to enhance GABA-mediated currents (Wafford and Ebert, 2008; Winsky-Sommerer, 2009; Feren et al., 2011). Some, like anesthetic agents (eg, barbiturates), act directly upon the GABA_A_R ion channel (Franks, 2008). Interestingly, anesthesia with GABA_A_R agonists (eg, propofol) can actually serve in the homeostatic response to SD in place of natural sleep recovery (Tung and Mendelson, 2004). Reciprocally, SD lowers the threshold to anesthesia induction, likely due to homeostatic changes in the GABA_A_R. Gamma hydroxybutyrate (GHB) used in the treatment of narcolepsy with cataplexy acts upon the GABA_B_R to consolidate sleep with low muscle tone during sleeping periods, such as to reduce narcoleptic attacks during the following waking period in humans and rodents (Xie et al., 2006; Vienne et al., 2010; Boscolo-Berto et al., 2012; Black et al., 2014). Moreover, GHB or its metabolite can alleviate the behavioral and physiological effects of sleep deprivation (Walsh et al., 2010). These results would also suggest that pharmacological effects upon the GABA_B_R, as upon the GABA_A_R, can mimic the homeostatic effects of sleep. However, such pharmacological effects are rarely cell specific and thus can affect both wake- and sleep-active cell groups, which as we show here would normally undergo differential homeostatic changes in their GABA receptors depending upon their state selective activity.

Sleep is regulated in a homeostatic manner (Borbély and Achermann, 1999), whereby SD is compensated for by enhanced NREM or slow wave sleep and delta EEG activity along with increased REM sleep. Whereas Orx neurons normally promote waking and prevent sleep including importantly REM sleep with muscle atonia (Mieda et al., 2004; Adamanitidis et al., 2007; Tsunematsu et al., 2011) MCH neurons normally enhance sleep including importantly REM sleep with muscle atonia (Verret et al., 2003; Jego et al., 2013; Konadhode et al., 2013; Tsunematsu et al., 2014). The reciprocal changes in the inhibitory GABA receptors and presumed excitability and activity of the Orx and MCH neurons seen here with SD could thus underlie the homeostatic response of decreased arousal and increased sleepiness during deprivation and increased sleep, including REM sleep, during recovery.

We conclude that expression and density of both GABA_A_ and GABA_B_ receptors increase on Orx neurons because of prolonged activity and reciprocally decrease on MCH neurons because of prolonged inactivity during SD. These reciprocal changes in excitability of the Orx and MCH neurons could decrease arousal and increase sleepiness along with sleep pressure during SD. During SR, the GABA receptors return to baseline presumably returning the excitability and activity of the Orx and MCH neurons to stable levels and thus restoring normal arousal while removing sleep pressure.

## Synthesis

The decision was a result of the Reviewing Editor Rae Silver and the peer reviewers coming together and discussing their recommendations until a consensus was reached. A fact-based synthesis statement explaining their decision and outlining what is needed to prepare a revision is listed below. The following reviewers agreed to reveal their identity: Thomas Kilduff, Patrick Fuller

In this interesting paper the authors report on factors associated with the expression/density of GABA receptors on orexin and MCH neurons. The manuscript provides evidence that GABA receptors on orexin neurons are homeostatically upregulated during sleep deprivation (SD). In contrast, in MCH neurons, GABA receptors are downregulated during SD.

The results indicate that effects on receptors are both a function of, and reflects differential responses to the antecedent behavioral state. These findings are consistent with existing cfos and in vitro electrophysiological data (and where it does not, the authors provide sound explanation). While poised to make an important contribution to the literature, the paper could be strengthened by addressing a few concerns. Primary among these concerns is that insufficient details are provided concerning several aspects of the study, as follows:

MAJOR ISSUES

It is not clear how the neurons were selected for luminescent measurements. For example, what criteria were used to select cells? How was selection bias avoided? And was the selector blinded to the condition of the animals?

Why are there so few neurons chosen for luminescent measurements? And do the authors have evidence that the 8-10 neurons counted for each animal were representative of the entire population in a particular animal?

The number of mice in each of the 4 experimental groups is not mentioned. This is a critical detail because the two SD groups were combined (I. 172-173) into a single group comprised of 11 mice. It would also be useful to know how many mice in each group were implanted for EEG recording.

The two GABA receptor antibodies used in this study are identified but no characterization of these antisera is presented nor a reference to the literature in which they have been characterized nor even mention of any immunostaining controls that the authors conducted in the absence of the primary antibody.

Was the immunostained material batch processed?

MINOR ISSUES

What is the difference between the grid size and counting frame? Please clarify.

Please clarify which mice in which groups had EEG headstages.

The figures aren't labelled (i.e. figure 1,2,3 etc) in the merged file.

Describing MCH neurons as 'sleep-promoting' is a bit of a stretch, if possibly inaccurate. Several in vivo optogenetic papers regarding MCH neurons and REM sleep have been published over the past few years. And the one thing that all of these studies reach consensus on is that activation of MCH neurons (using physiological stimulation paradigms) increases REM sleep epoch, and rather modestly at that.

The following point might be amplified: Work from Barbara Jones' lab has also shown [recording in head-fixed prep with juxtacellular labeling post hoc] that MCH neurons fire maximally during REM sleep (paradoxical sleep), and only occasionally during NREM (SWS) sleep.

Electrophysiologically, 100% orexin neurons tested respond to muscimol (Eggermann 2003, Xie 2006).

Why do the authors think that the number of orexin neurons expressing GABA-A receptors is so low when measured using immunohistochemical methods?
